# Butter, Margarine, Vegetable Oils, and Olive Oil in the Average Polish Diet

**DOI:** 10.3390/nu11122935

**Published:** 2019-12-03

**Authors:** Hanna Górska-Warsewicz, Krystyna Rejman, Wacław Laskowski, Maksymilian Czeczotko

**Affiliations:** Department of Food Market and Consumer Research, Institute of Human Nutrition Sciences, Warsaw University of Life Sciences, 02-787 Warsaw, Poland; krystyna_rejman@sggw.pl (K.R.); waclaw_laskowski@sggw.pl (W.L.); maksymilian_czeczotko@sggw.pl (M.C.)

**Keywords:** fats and oils, energy intake, nutrient intake, food sources

## Abstract

The main aim of this study was to identify the sources of energy and 25 nutrients in fats and oils in the average Polish diet. We analyzed energy, total fat, saturated fatty acids (SFAs), monounsaturated fatty acids (MUFA), polyunsaturated fatty acids (PUFA), cholesterol, protein, carbohydrates, nine minerals, and nine vitamins. We included five sub-groups: butter, vegetable oils, margarine and other hydrogenated vegetable fats, olive oil, and other animal fats. The basis for our analysis was data from the 2016 household budget survey, conducted on a representative sample of the Polish population (36,886 households, *n* = 99,230). We used the cluster analysis to assess the impact of socio-demographic and economic factors on the volume of fats and oil consumption and on the share of particular products in the supply of energy and nutrients. Our findings indicated that fats and oils contributed 32.9% of the total fat supply, which placed these products in first position among main food groups. Meat and its products ranked second (30.8%) in the total fat supply, while milk and dairy products, including cream (13.4%), were the third food group. The second position in the total fat supply was taken by meat and its products (30.8%), and the third place was taken by milk and dairy products, including cream (13.4%). The supply of fatty acids from fats and oils varied and ranged from 45.6% for PUFA to 31.5% for MUFA to 27.8% for SFA. The supply of cholesterol was at the level of 8.3%. Our research has proven that fats and oils are an important source of vitamin E, providing almost half of the daily supply of this vitamin to the average Polish diet. The supply of vitamin A and D equaled 16–18% of their total daily intake. In the cluster analysis, we identified five clusters that differed in the consumption of butter, oils, margarine and other vegetable fats, olive oil, and other animal fats. The variables with most differentiating clusters were: education level, income (in quintile groups of households), degree of urbanization of the place of household residence, and socio-economic type of the household. Our results indicate a high share of fats and oils in the total fat supply and should be used to evaluate the diets from a nutritional and health point of view.

## 1. Introduction

Fat as a macronutrient is needed for humans in relatively large amounts as a source of energy and fatty acids, a heat conserver, a component of cell walls, and a transport vehicle for absorption fat-soluble vitamins A, D, E, and K, which serve as a way of insulating the body and as a shock absorber [1]. 

Fats and fatty acids are essential nutrients, but the amount and type of fat consumed have differential effects on health and important implications for chronic disease prevention and treatment [2,3,4,5,6,7,8,9]. Studies indicate that the dietary fats have distinct effects on the risk of cardiovascular diseases (CVDs) and incidence of other major chronic diseases, including type 2 diabetes, cancer, multiple sclerosis, and respiratory diseases [8,10]. The composition of fat in relation to the proportion of saturated fatty acids (SFAs), polyunsaturated fatty acids (PUFA), and monounsaturated fatty acids (MUFA) is taken into account in the analyses. SFAs are involved in the development of CVDs [8] and coronary heart disease (CHD) [3,4,5,6,7]. Higher intake of saturated and trans-fats was associated with increased risk of CHD, whereas a higher intake of monounsaturated and polyunsaturated fats was associated with a decreased risk [4]. This is particularly important in the regions where intake of total fat and SFAs are high [11]. As a result, to prevent chronic diseases, such as CHD, most dietary recommendations focus on the reduction of saturated fatty acids intake [11,12,15]. However, the risk of CHD associated with SFAs varies from no association to a significantly important risk [3]. WHO/FAO (World Health Organization/Food and Agriculture Organization of the United Nations) expert recommendations for the total fat intake vary between 15 and 30% of the dietary energy, and at least 20% of the total energy delivered from total fat is consistent with good health. For each type of fat, the recommendations for the share of dietary energy are as follows: SFAs <10%, PUFAs 6–10%, of which n-6 PUFAs 5–8% and n-3 PUFAs 1–2%, and trans-fatty acids <1% [12].

The category of fats and oils includes butter, vegetables oils, margarine, olive oil, and other animal and vegetable fats. Studies suggest that butter consumption increases plasma cholesterol and HDL (High Density Lipoprotein) cholesterol concentration, which ensures that total/HDL cholesterol ratio remains mostly unchanged [13]. Other results indicate relatively small or neutral associations of butter consumption with mortality, CVD, and diabetes [2], as well as cancer mortality [14]. Butter, added fats and oils, and meat and meat products are the three main sources of the total fat and SFA in diets. This is the subject for systematic research due to the fact that the majority of diets in the European population is characterized by increased SFA intake. Data on fatty acid consumption in 24 European countries showed a large variation in fat intake from 28.5 to 46.2% of total energy supply, (SFA from 8.9 to 15.5% and PUFA from 3.9 to 11.3%) [11].

Based on these arguments, the importance of the total fat intake, as well as individual products from the category of fats and oils in the diet, should be indicated. Therefore, the purpose of our research was to identify food sources of energy and 25 nutrients from the fats and oils category based on the data from the 2016 household budget survey. We also analyzed the impact of socio-demographic and economic factors on the volume of fat and oil consumption, as well as the level and structure of energy and nutrient supply from this food category. This paper is the fifth consecutive article based on the same methodology concerning the sources of energy and nutrients in the average Polish diet. So far, we have analyzed the food sources of protein and amino acids [15], as well as three groups of food categories (meat, seafood and its products [16], milk and dairy products [17], and cereal products [18]) as sources of energy and nutrients.

## 2. Methods

### 2.1. Study Overview

In this study, we analyzed the sources of energy and nutrients from fats and oils in the average Polish diet. The detailed list of these nutrients includes: total fat, fatty acids (saturated fatty acids (SFA), monounsaturated fatty acids (MUFA), polyunsaturated fatty acids (PUFA)), cholesterol, carbohydrates, protein, 9 minerals (calcium, phosphorus, sodium, potassium, magnesium, iron, zinc, copper, and iodine), and 9 vitamins (vitamin A, vitamin D, vitamin E, thiamin, riboflavin, niacin, vitamin B6, folate, and vitamin B12). We presented the percentage of total fat contribution from other main food categories: meat and meat products, milk and dairy products (including cream), cereal products, snacks and sweets, eggs, seafood, vegetables, and fruits. We also analyzed the impact of socio-demographic and economic factors on the volume of fat and oil consumption and the share of particular products in the supply of energy and nutrients.

The overall test procedure was as follows:

1. Two-stage random selection of the representative sample of the households (36,886 households), carried out by the Central Statistical Office;
2. Data collection on quantity of purchase and consumption of food products in 91 sub-groups (in grams, kilograms, liters) per month per household (Central Statistical Office);
3. Conversion of consumption quantity into one person per month in each household (in grams, kilograms, liters)—our calculations;
4. Conversion of consumption data into energy and nutrients content (in kcal, g, mg, µg per day) in 91 sub-groups of consumed food products in each household—our calculations;
5. Calculation of the average energy and nutrients supply in sub-groups in kcal, g, mg, µg per day per person in all households—our calculations;
6. Calculation of the energy and nutrient contribution from each sub-group (in %) to the average Polish diet—our calculations;
7. Analysis of impact of socio-demographic and economic factors on the level and structure of energy and nutrient supply from fats and oils—our calculations.

Additional information on the methodology of the Household Budget Survey has been provided in our previous publications on meat, seafood and its products, cereal products, and milk and dairy products, as well as food sources of protein [15,16,17,18].

### 2.2. Sample Selection Method

We used data from the household budget survey (HBS), organized and conducted by the Central Statistical Office (CSO), Social Surveys and Living Conditions Statistics Department. The HBS is a representative method of examining households across Poland in terms of many issues related to the consumption of goods and services and their living conditions. In 2016, 36,886 households participated in the survey, which constituted 99,230 people. The procedure for selecting households was a two-stage process, which results from the draw, in the first stage, of the areas survey points, and in the second of specific households in each area survey point. In 2016, during the first stage, 1586 area points were drawn, including 911 area points in cities and 665 in rural areas. Thereafter, households were drawn.

In each household, the volume of purchase of food products to be eaten in the household was recorded in terms of volume (in grams, kilograms, pieces, liters) and value (in Polish zlotys) in a “Household Budget Diary” for one month. Additional information was obtained through detailed interviews in each household based on the “Household’s Statistical Sheet” and conducted by the employees of the regional statistical offices. We used the data obtained by CSO from 36,886 households to calculate the volume of food consumption per person per month [23,24].

In 2016, the share of women in the surveyed population was 52.4%. In terms of age, the distribution of the studied population was as follows: 25–34 years (12.6%), 35–44 years (13.8%), 45–54 years (12.5%), 55–64 years (15.8%), and 65 years and above (17.1%). In the study, according to the CSO methodology, four main types of households were included: employees (*n* = 17,877 households, *n* = 55,799 people), farmers (*n* = 1689, *n* = 6481), self-employed (*n* = 2500, *n* = 7970), and pensioners (*n* = 13,323, *n* = 25,195). In terms of the number of persons in a household, the structure of the surveyed population was as follows: one-person households (*n* = 7590, *n* = 7590), two-person households (*n* = 12,085, *n* = 24,170), three-person households (*n* = 7300, *n* = 21,900), four-person households (*n* = 6130, *n* = 24,520), five-person households (*n* = 2363, *n* = 11,815), and six or more-person households (*n* = 1418, *n* = 9235).

### 2.3. Food Grouping

Data on food consumption in households concerned 91 food sub-groups, which, for the purpose of our analysis, were divided into 13 main categories, i.e., meat and meat products; cereal and grain products; milk and dairy products; sugar, sweets, and snacks; vegetables and vegetable products; fruits and fruit products; eggs; seafood; coffee, tea and cacao; nonalcoholic beverages; alcoholic beverages; and fats and oils. A detailed classification has been published in our previous publications [15,16,17,18]. For the purposes of this article, we have considered fats and oils containing 5 sub-groups:

- Butter;
- Vegetable oils;
- Olive oils;
- Margarine and other vegetable fats;
- Other animal fats.

For this classification, we used a food classification scheme published in literature [19,20,21], with definitions from FAO [22] and CSO [23,24]. These classifications in some cases differ in the recognition of particular types of animal and vegetable fats. For example, FAO defines animal fats as a group consisting of: slaughter tissue fats, rendered fats (lard, in Poland, is traditionally obtained from pigs and poultry, especially from goose), and oils from fish and marine mammals [22]. According to the CSO, animal fats include butter, bacon, lard, tallow, jowl, and others (raw and rendered). Butter is classified as fresh, melted, and salted and contains up to 20% of vegetable oil. Margarine and other vegetable fats include margarine, vegetable butter, mixtures of butter, and so-called “vegetable butter”, vegetable oils, olive oil, and others [24].

### 2.4. Data Analysis

Having quantitative data on the amount of food consumption, we converted them for the supply of energy and nutrients. For this conversion, we applied the latest edition (4th) of the Polish “Nutritive Value Tables for Foods and Meals”) [25] and the R software environment for statistical computing (v3.0.2) (The R Foundation for Statistical Computing, Vienna, Austria 2018) [26,27,28]. In this way, we received data on the supply of energy and nutrients in each household. Thereafter, we calculated the average energy supply and the average supply of individual nutrients, which was expressed in kcal, g, mg, µg per person per day. This allowed us to determine the share (in %) of each sub-group of food in the supply of energy and nutrients to the average Polish diet.

Subsequently, we analyzed the impact of socio-demographic and economic factors on the consumption of fats and oilsm as well as the level and structure of energy and nutrients supply from fats and oils. For this purpose, we used cluster analysis [29,30,31], using the Neural Networks module in the Statistica 13.3 (Copyright 1984–2917, TIBCO Software Inc., Palo Alto, CA, USA) and the Kohonen Neural Network [32]. We have divided the sample population into 5 clusters based on 14 factors, characterizing households: education level, income (quintile group), degree of urbanization of the place of household residence, socio-economic type of household, size of the village, usage of agricultural land, self-assessment of financial situation, number of people in the household, region, family life phase, self-assessment of nutrition in household, age, sex, and month of participation in the survey. We calculated the Cramer’s correlation for each feature.

## 3. Results

### 3.1. Contribution of Food Categories to Total Fat Intake

Fats and oils ranked as the first among sources of total fat in the average Polish diet, providing almost 1/3 of the supply of total fat (Table 1). The second main food category was meat and its products, and the third was milk and dairy products. In total, these three main groups of products provide over 3/4 of the daily total fat intake in the average Polish diet.

### 3.2. Fats and Oils as Sources of Energy and Nutrients

#### 3.2.1. Fats and Oils as Sources of Energy

Fats and oils provide 298 kcal to the average Polish diet (Table 2). Given that the daily energy supply is 2261 kcal, fat and oil contribution equals 13.2%. Vegetable oils come first in the energy supply, delivering 128 kcal (5.7%), followed by butter, margarine, and other vegetable fats. Total fat intake in the average diet (96.9 g as shown in Table 1) provides 872 kcal, which is 38.6% of the dietary energy supply. This means that the share of fat in the supply of dietary energy significantly exceeds the recommendation.

#### 3.2.2. Fats and Oils as Sources of Nutrients—General Overview

Our analysis concerns the supply of 25 nutrients from fats and oils in the average Polish diet. We are aware that this food category provides very small quantities of B vitamins and minerals. However, we have presented the results for the same nutrients that were included in our previous analyses for the sake of consistency. Fats and oils provide 32.9% of the total fat supply (Table 3). The supply of fatty acids varies and ranges from 45.6% for PUFA to 31.5% for MUFA, to 27.8% for SFA. Fats and oils supply 0.92 g of omega-3 fatty acids, which is 39.9% of their total intake in the average Polish diet (amounting to 2.4 g/person/day). In the case of omega-6 PUFA, fats and oils deliver 6.5 g, which is 44.4% of their total intake in the average Polish diet (amounting to 14.6 g/person/day). The supply of cholesterol from fats and oils is at the level of 8.3%. From among the examined vitamins, fats and oils are an important source of vitamin E, providing almost half of the average daily supply of this vitamin. The supply of vitamins A and D amounts to 16–18% of their total daily supply.

#### 3.2.3. Fats and Oils as Sources of Nutrients—Detailed Analysis

For a detailed analysis, we included those nutrients for which fats and oils are an important source in the structure of the average Polish diet. In Table 4, we present the share of butter, vegetable oils, olive oil, margarine, and other vegetable fats, as well as other animal fats, in contribution to total intake of fat, SFA, MUFA, PUFA, cholesterol, vitamin A, vitamin D, and vitamin E.

In the structure of the total fat supply in the average Polish diet, the share of fats and oils equaled 32.9%, with vegetable oils, margarine, and butter having the largest share. The supply of SFA from fats and oils was 9.7 g (Table 3), which accounted for 27.8% of the total supply. Butter was the main source of SFA, accounting for 13.9% of the total daily supply. The supply of MUFA was 11.80 g (Table 3), i.e., 31.5% of the total supply with vegetable oils, having the largest share. In the case of PUFA, fats and oils supplied 8.2 g in the average Polish diet (Table 3), accounting for over 45% of the total supply.

#### 3.2.4. Fats and Oils as Sources of Nutrients—Summary

**Figure 1:**
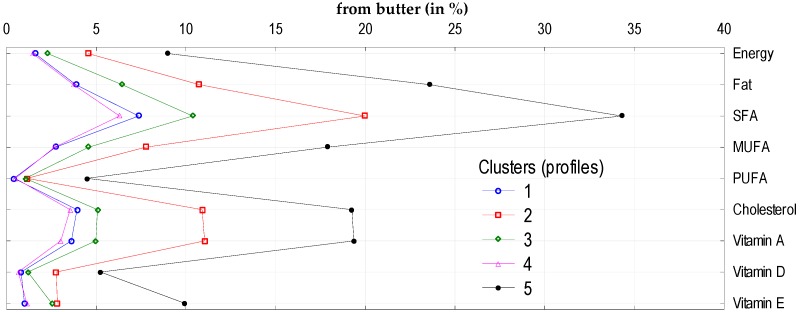
Cluster analysis: Supply of energy and analyzed nutrients (in %) from butter to diets in individual clusters. 1, 2, 3, 4, 5—number of clusters, characteristics of clusters are presented in Table 6.

**Figure 2:**
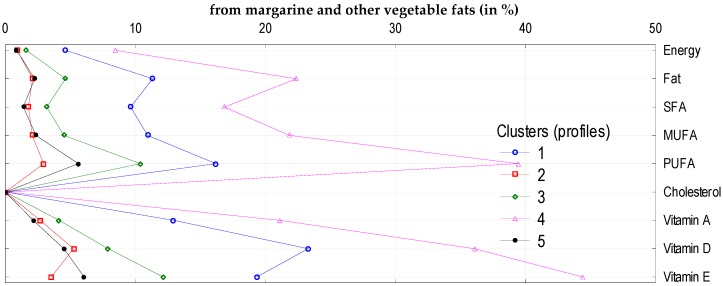
Cluster analysis: Supply of energy and analyzed nutrients (in %) from margarine and other vegetable fats to diets in individual clusters.

**Figure 3:**
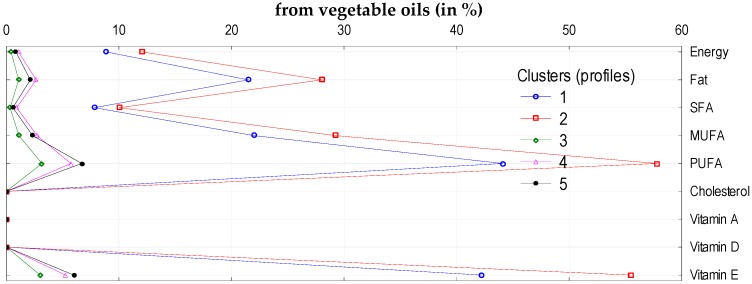
Cluster analysis: Supply of energy and analyzed nutrients (in %) from vegetable oils to diets in individual clusters.

To analyze the impact of socio-demographic and economic characteristics of households on the volume of consumption of five sub-groups of fats and oils (butter, vegetable oils, olive oil, margarine and other vegetable fats, and other animal fats), we identified five clusters. The following factors had the greatest impact on the volume of consumption of these products and the supply of energy and nutrients: education level, income (in quintile groups of households), degree of urbanization of the place of the household residence, and socio-economic type of household (Table 5). The clusters differed in terms of monthly butter consumption per capita (lowest consumption: 0.13 kg per person per month in Cluster 4, highest 0.78 kg per person per month in Cluster 5). This mainly affected the differences in the share of butter in total fat and SFA supply (Figure 1). The average consumption of margarine and other vegetable fats amounted to 0.38 kg per person per month and was the highest in Cluster 4 (0.92 kg) and the lowest in Cluster 5 (0.10 kg) (Table 6). This resulted in the highest contribution of margarine and other vegetable fats in the supply of PUFA and vitamin E (Figure 2). Vegetable oil consumption was 0.5 L per person per month and varied depending on the clusters: The highest was found in Cluster 2 (1.05 L) and the lowest in Cluster 3 (0.05 L) (Table 6). This largely affected the supply of PUFA and vitamin E, as was the case for margarine and other vegetable fats (Figure 3).

## 4. Discussion

Fats and oils are a significant source of energy in the average Polish diet, as are the total fats, MUFA, PUFA, SFA, and cholesterol. The purpose of our study was to determine the supply of energy and nutrients from the fats and oils category, taking into account five sub-groups, including butter, vegetable oils, olive oils, margarine and other vegetable fats, and other animal fats. We compared our results with those from other countries, including the United States (2003–2006) [21], Belgium [33], Ireland [34], Australia [35,36], Spain [37], Denmark [38], and New Zealand [39], as well as with population nutrient intake goals [12].

Our research indicated that fats and oils provided 13.2% of the total energy supply to the average Polish diet. Of the five sub-groups of fats and oils studied, vegetable oils were the most important in energy supply (5.7%), followed by butter (3.3%) and margarine and other vegetable fats (3.2%). The data obtained for the average Polish diet are much higher compared to other studied populations [21,35,39,42]. For example, in the average American diet, vegetable oils and other fats provided 3.6% of the daily energy supply, while butter and margarine provided 2.2% [21]. Similar results were obtained for the Australian diet (the share of oils and fats in the energy supply amounted to less than 4%, of which margarine accounted for about 2.5%) [35] and the New Zealand diet (the share of total fat in the energy supply was 3.3%, of which the share of butter and margarine was 3%) [39]. There was a higher share of fats and oils in the energy supply in the Polish diet, but was only half as much, compared to the average diet recorded in Denmark. In the Danish diet, the share of total fat in the energy supply as 7% [42].

The high share of energy from fats and oils determines subsequent results, showing the share of this category of food products in the total fat supply, SFA, MUFA, and PUFA. In the supply of total fat, fats and oils accounted for almost 33% of the total contribution. The largest share had oils (almost 14%), followed by margarine and other plant fats and butter (each of these sub-groups were almost 8.5%). In the structure of the total fat supply in the average Belgian diet, the group of fats and oils provided 27.1% of total fat, including margarine at 11.8%, butter at8.9%, vegetable oils at3.3%, and deep frying fats at2.6% [33]. Studies conducted in the American population indicated the share of oils and other fats in the total fat supply at the level of 9.6%, while margarine and butter accounted for 6.4% of the total fat supply [21]. Similar figures were recorded for the New Zealand diet: The share of fat in total fat supply was 10.2%, of which 9.3% was recorded for butter and margarine [39]. In the Australian diet, fats and oils provided less than 12% of the total dietary fat [35], while in the Danish diet it was 19% [42].

Fats and oils provided 27.9% of SFA to the average Polish diet. Butter had the largest share, accounting for 13.9% of the supply, followed by vegetable oils (4.8%), as well as margarine and other vegetable fats (4.5%). Similar results were obtained for the average Belgian diet. The share of fats and oils in the supply of SFA was 25%, including butter (13%) and margarines (8.3%) [33]. In the average American diet, the share of vegetable oils and other fats in the supply of SFA was 8.9%, while margarine and butter contributed 6.3% [21]. Similar results were obtained for the Australian diet: The share of oils and fats in the supply of saturated fat was 9% [39]. In the New Zealand diet, the share of edible fats in total fat supply was 8.9%, of which butter and margarine accounted for 8.4% [39]. A higher percentage of fats in the SFA supply was recorded in the Danish diet at 14% [42].

Our research indicated that fats and oils contributed 31.5% of MUFA with the largest share of oils (14.2%), margarine and other plant fats (8.1%), and butter (6.1%). The structure of the MUFA supply in the American diet was different: 10.5% of oils and other fats and 6.4% of margarine and butter [21]. A lower supply of MUFA from fats and oils was recorded for the Australian diet (less than 12.5%), including about 2.5% for milk fats and about 8% for margarine [35]. In the average Danish diet, the share of fat in the supply of MUFA was 19% [42], while in the New Zealand diet it was 11% [39].

In the supply of PUFA to the average Polish diet, fats and oils accounted for almost 46% of all-day contribution. Vegetable oils were the most important (almost 30%), followed by margarine and other plant fats (14.1%). A lower share of fats and oils in the supply of PUFA was found in the average Belgian diet at 32.4%, including margarines at 22.8%, vegetables oils at 3.8%, deep frying fats at 3.3% and butter at 2% [33]. Similar results were obtained for the average Danish diet, in which fats provided 32% of PUFA [42]. In the average Australian diet, the share of fats and oils in the supply of PUFA was about 22%, of which margarine accounted for almost 20% [35]. On the other hand, in the American diet, oils and other fats provided 11.7% of PUFA, while margarine and butter provided 7.4% [21]. From the nutritional point of view, it would be valuable to calculate the intake of trans-fatty acids in the Polish population. We are not able to show in our study these fat intakes, as the Polish food composition and nutritional value tables do not give their contents in food products. However, the 30 year-long process of creating a modern food market in Poland was accompanied by changes in the food consumption pattern, which is typical for countries in a period of dynamic economic growth. The changes consisted mainly of an increase in the consumption of meat, fats, and highly processed foods [40]. As a result, the structure of consumption of fats and oils changed. In particular, the consumption of fats and oils of vegetable origin increased 2.8 times and amounted to 24.5 kg/person in 2018 (at the level of food balances based on CSO data). It can be assumed (using our own calculations based on CSO data) that, in this amount, about 9.7 kg are fats used in highly processed foods, including hydrogenated vegetable oils containing trans-fatty acids. Some results indicated that the average intake of trans-fatty acids in Poland is 2.8–6.9 g per day. This means that the intake exceeds the dietary recommendations [41,42], according to which the content of these fatty acids in the daily diet should not exceed about 2 g (1% of the energy value of the diet) [12].

According to our results, fats and oils provided 47.6% of vitamin E, of which 27.7% was delivered from oils and 16.3% from margarine and other vegetable fats. Similar data were obtained for the average Spanish diet, for which oils and fats were the main contributors (45.7%) to the vitamin E intake [37]. A lower fat content in the supply of this vitamin was found for the Danish and New Zealand diets (24% and 14.2%, respectively) [39,42]. The results for the average Polish diet and their comparison with other countries are worrisome for nutrition and health reasons. As shown in Table 2, fats and oils constituted 1/3 of the total fat supply in the average Polish diet. Therefore, twice as much fat was derived from invisible fats with the highest proportion from the meat and meat products group (31%) and then from the dairy products group (14%, including cream). The consequence of such a fat consumption structure is a high SFA content of 35 g in the diet, which is unfavorable for health, while the MUFA content was 37 g and the PUFA was 18 g (as shown in Table 4). This amount of SFA constitutes 13.9% of the daily energy supply in the average Polish diet, while WHO/FAO population nutrient intake goals indicate the share of SFA to be below 10% [12]. Recommended dietary allowances in Poland are even more restrictive, as it is recommended to have 10% of SFA shared in the daily energy supply, only for children aged 1–9 years, and almost twice as low as 5–6% for other population groups. Furthermore, it was stated that the recommended daily intake of SFA should be “as low as reasonably achievable in a nutritionally appropriate diet” [43]. In the United States, the National Heart, Lung, and Blood Institute (NHLBI), the American College of Cardiology (ACC), and the American Heart Association (AHA) advise lifestyle management recommendations for adults who have elevated low-density lipoprotein cholesterol (LDL–C) intake (which concerns 33.5% of the population). The intension is to reduce the percentage of calories from saturated fat to achieve 5 to 6% of calories from saturated fat [44]. Randomized controlled trials, that lowered the intake of dietary saturated fat and replaced it with polyunsaturated vegetable oil, reduced CVDs by ca. 30%, similar to the reduction achieved by statin treatment. Prospective observational studies in many populations showed that a lower intake of SFA coupled with a higher intake of PUFA and MUFA is associated with lower rates of CVD and other major causes of death and all-cause mortality [45]. Improving dietary fat quality by replacement of SFA with n-6 and n-3, PUFA has also a significant influence on the reduction of risk of sudden cardiac death [46]. Dietary guidelines for Americans in 2015–2020 assumed a shift from solid fats to oils to use oils rather than solid fats in food preparation where possible. This refers to the points: To use vegetable oils instead of solid fats (butter, stick margarine, shortening, lard, coconut oil) in cooking; to increase the consumption of products containing oil naturally; and to choose other foods, such as salad dressings and spreads [47].

It should be underlined that the share of PUFA in the energy supply in the average Polish diet equaled 7.13% and was close to the lower limit recommended by WHO/FAO experts (6–10%) [12]. An adequate dietary intake of docosahexaenoic acid (DHA, 22:6, n-3) is particularly important for pregnant and lactating women, children, and adolescents. As the consumption of fish and seafood in Poland is very low, dietary supplementation with DHA is recommended [43,48,49]. However, research carried out in various countries, where the consumption of fish and seafood is higher, also clearly shows this problem. Data from 2003–2008 NHANES (National Health and Nutrition Examination Survey) indicated that US adults did not meet the recommended levels for fish and omega-3 fatty acid intake [50]. In the case of women, NHANES survey results from the period 2001–2014 showed that a majority of US women of childbearing-age and pregnant women consumed significantly lower amounts of seafood than what the DGA (The 2015–2020 Dietary Guidelines for Americans) recommends, which subsequently leads to low intakes of EPA (eicosapentaenoic acid) and DHA (docosahexaenoic acid). Moreover, diet supplementation did not eliminate the deficiencies of these nutrients [51].

The third unfavorable feature concerning fat consumption in Poland was too high a proportion of energy supplied by fats in the average diet compared to the recommendations. It amounted to 38.68% of the daily energy supply, i.e., exceeding WHO/FAO recommendations by almost 9%. This is not only the case in Poland, but it poses the most crucial threat to public health globally, even in developing countries, following so-called western diets and facing different double burden issues [52,53,54]. An increase in the number of overweight and obese people was observed in Poland as well as in other European countries [55,56,57,58,59,60]. In Poland, in 2014, over 62% of adult men weighed too much (44% were overweight and 18% were obese). Among women, the problem concerned over 46% (30% were overweight and 16% were obese) [61]. Among dietary factors, both total energy supply and fat intake are significantly correlated with body mass index. Moreover, increased intake of fat energy is associated with a greater per unit increase in body mass than the increased intake of energy from non-fat sources [1]. However, according to a recent Cochrane review [62], the proportion of energy from fat in food consumed, and its relation to body weight, is not clear. On the base of RCT (randomised controlled trias) results (approx. 54,000 participants), the consistent evidence in adults of a small weight-reducing effect of eating a smaller proportion of energy from fat was found. Simultaneously included cohort studies in children and adults most often did not suggest any relationship between total fat intake and later measures of weight, body fatness, or change in body fatness. However, there was a suggestion that lower fat intake was associated with smaller increases in weight in middle-aged but not elderly adults and in a change in BMI in the highest validity child cohort. Another systematic review and meta-analysis of prospective cohort studies (approx. 89,800 participants) found epidemiological research that pointed to no significant difference in CHD mortality and total fat or saturated fat intake. These findings do not support the present dietary fat guidelines [63].

Therefore, further research is needed and, at the same time, action to improve public health is important. In Poland, a four-year National Health Program 2016–2020 is being implemented [64]. The operational objective of the program is to improve dietary patterns, nutritional status, and physical activity of the population. This program is seen as a very important step in the prevention of obesity due to the planning of comprehensive activities supported by the state budget. In previous health programs, there was no specific reference to the fight against overweight and obesity in the Polish society, including children [65].

The 2016 HBS representative sample size, a consistent approach to classifying food products, and HBS methodology to record purchased and consumed food are strengths of the presented study. However, there are some limitations, particularly the reliance on self-recording of information on consumption in a diary. This can lead to an under- and/or overestimation of consumption data, even though HBS uses well-established procedures to control all recordings. The current edition of “Nutritive Value Tables for Foods and Meals” (4th ed., 2017) includes new products and technological modifications, which may cause difficulties in the comparison of current results with data from earlier years. However, these limitations of the HBS survey are rather typical. Nevertheless, household budget surveys are the only representative method for systematic data collection regarding food consumption in the Polish population.

## 5. Conclusions

Our study indicated that fats and oils are an important food category in the average Polish diet, delivering 31.8 g of fat, which is almost 1/3 of the total daily fat supply. Another 30% of dietary fat comes from meat and meat products. Milk products are the third food category among the sources of fat in the Polish diet. Together, these three food categories contribute 77% of the total intake of this nutrient. The share of fatty acids derived from fats and oils in the average diet is over 45% for PUFA, 31.5% for MUFA, and almost 28% for SFA. The sub-group of vegetable oils dominates in the fat supply (14%), as is similarly the case for PUFA (29%) and MUFA (14%). However, butter has the largest share in the supply of SFA (14%). Fats and oils provide 298 kcal of energy in the average Polish diet, which means 13.2% of the total energy supply. It is slightly over 1/3 of the total amount of 38.6% of the dietary energy supply from fat. The fact that the intake of fat and saturated fat is higher than recommended is likely to contribute to chronic diseases. On the one hand, the results of our research should be useful to intensify initiatives of the government agencies and health professionals in the frame of the national health program and, on the other, it can be a tool for assessing the effectiveness of actions taken.

## Figures and Tables

**Table 1:** Main food categories as the sources of total fat supply in the average Polish diet (in % of total fat contribution).

Specification	Total Fat Contribution	
	In g	In %
All food categories	96.9	100.0
fats and oils	31.8	32.9
meat and meat products	29.9	30.8
milk and dairy products (including cream)	13.0	13.4
milk and dairy products (without cream)	10.9	11.3
grain products	9.6	9.9
snacks and sweets	6.1	6.3
eggs	2.3	2.3
vegetables	2.0	2.0
seafood	1.2	1.2
fruits	1.1	1.1
others	0.1	0.1

**Table 2:** Sources of energy contribution from fats and oils to the average Polish diet (in kcal and % of total energy contribution).

Specification	Energy	
	In kcal	In %
Average daily supply in kcal	2261	100.0
Contribution of fats and oils	298	13.2
butter	74	3.3
olive oil	4	0.2
vegetable oils	128	5.7
margarine and other vegetable fats	73	3.2
other animal fats	19	0.8

**Table 3:** Contribution of nutrients from fats and oils to the average Polish diet (in % of total contribution).

Specification	Average Polish Diet	Contribution from Fats and Oils	
		In g, mg or µg	In %
**Macronutrients**			
total fat	96.9 g	31.84 g	32.9
SFA	34.8 g	9.68 g	27.8
MUFA	37.4 g	11.80 g	31.5
PUFA	17.9 g	8.17 g	45.6
including			
omega-3 PUFA	2.4 g	0.92 g	39.9
omega-6 PUFA	14.6 g	6.50 g	44.4
cholesterol	316.0 mg	26.20 mg	8.3
protein	77.9 g	0.17 g	0.2
carbohydrates	270.4 g	0.11 g	0.0
Vitamins			
vitamin A	1194.6 µg	195.43 µg	16.4
vitamin D	4.6 µg	0.82 µg	17. 8
vitamin E	13. 5 mg	6.41 mg	47.6
thiamin	1.3 mg	0.004 mg	0.3
riboflavin	1.6 mg	0.006 mg	0.4
niacin	16.2 mg	0.02 mg	0.1
vitamin B6	1.8 mg	0.01 mg	0.4
folate	275.0 µg	0.30 µg	0.1
vitamin B12	4.5 µg	0.005 µg	0.1
Minerals			
calcium	644.1 mg	2.32 mg	0.4
phosphorus	1160.2 mg	1.74 mg	0.2
sodium	3863.8 mg	13.52 mg	0.4
potassium	2617.9 mg	3.40 mg	0.1
iron	10.3 mg	0.03 mg	0.3
magnesium	267.3 mg	0.19 mg	0.1
iodine	154.6 µg	0.74 µg	0.5
copper	1.1 mg	0.01 mg	0.5
zinc	9. 8 mg	0.01 mg	0.15

MUFA—monounsaturated fatty acids, PUFA—polyunsaturated fatty acids, omega-3 PUFA—omega-3 polyunsaturated fatty acids, omega-6 PUFA—omega-6 polyunsaturated fatty acids.

**Table 4:** Contribution of selected nutrients from fats and oils to the average Polish diet (in %).

Specification	Contribution (In %) from					
	Total Category of Fats and Oils	Butter	Olive Oil	Vegetable Oils	Margarine and Other Vegetable Fats	Other Animal Fats
Total fat	32.9	8.3	0.5	13.7	8.4	2.0
SFA	27.8	13.9	0.2	4.8	6.5	2.3
MUFA	31.5	6.1	0.8	14.2	8.1	2.3
PUFA	45.6	1.2	0.4	29.1	14.1	0.9
Cholesterol	8.3	7.6	0.0	0.0	0.0	0.7
Vitamin A	16.4	7.5	0.0	0.0	8.8	0.0
Vitamin D	17. 8	1.9	0.0	0.0	15.8	0.1
Vitamin E	47.6	2.8	0.5	27.7	16.3	0.3

**Table 5:** Dependence of cluster analysis on socio-demographic and economic factors.

Factors	Cramer Correlation
education level	0.135
income (in quintile groups)	0.117
degree of urbanization of the place of the household residence	0.116
socio-economic type of household	0.103
size of the village	0.096
usage of agricultural land	0.083
self-assessment of financial situation	0.078
number of people in household	0.069
region	0.065
family life phase	0.061
self-assessment of nutrition in household	0.057
age	0.043
sex	0.040
month of participation in the survey	0.036

**Table 6:** Cluster description.

	Cluster 1	Cluster 2	Cluster 3	Cluster 4	Cluster 5	Whole Population
number of households in clusters	9853	9315	7329	5497	4892	36,886
number of people	29,124	23,973	19,063	15,010	12,060	99,230
Structure (%) by education level						
junior high school, primary	17.0	11.8	12.1	17.8	8.2	13.7
basic vocational	38.2	27.8	26.1	40.0	22.0	31.3
secondary and post-secondary	31.1	34.3	33.1	30.3	34.4	32.6
higher	13.7	26.0	28.8	12.0	35.5	22.4
Structure (%) by income (in quintile groups)						
1 (20% of persons with the lowest income)	26.5	15.9	16.8	28.2	10.4	20.0
2	24.5	18.3	17.9	22.4	14.6	20.0
3	20.2	20.9	18.6	20.0	20.1	20.0
4	16.8	22.6	20.1	16.5	25.3	20.0
5 (20% of persons with the highest income)	12.1	22.4	26.6	12.8	29.7	20.0
Structure (%) by degree of urbanization						
densely populated area	26.4	37.7	40.1	29.6	48.6	35.4
intermediate populated area	23.6	23.3	21.8	23.6	21.7	22.9
sparsely populated area	50.0	39.0	38.1	46.8	29.7	41.7
Average monthly consumption (in kg/L per person)						
butter (in kg)	0.15	0.46	0.21	0.13	0.78	0.31
margarine and other vegetable fats (in kg)	0.56	0.12	0.19	0.92	0.10	0.38
olive oil (in liters)	0.01	0.02	0.03	0.01	0.03	0.02
vegetable oils (in liters)	0.75	1.05	0.05	0.10	0.09	0.50
other animal fats (in kg)	0.08	0.07	0.10	0.07	0.05	0.08
